# The Story of the Insane from Year to Year

**Published:** 1900-09-15

**Authors:** 


### THE STORY OF THE INSANE FROM YEAR TO YEAR.

Twelfth Series.

### THE WARWICK COUNTY ASYLUM.

On January 1st, 1898, this asylum contained 970 patients, and the number had risen to 986 on December 31st. There were 188 first admissions, 30 readmissions, 48 recoveries, 25 discharged relieved, and 113 deaths. The recoveries stand at 26·8 of the admissions, and the deaths 11·7 of the average number resident. Of the deaths, 56 were caused by cerebral and spinal diseases, 14 by consumption, 10 by pneumonia, and 2 by bronchitis. Of the admissions, 23 owed their insanity to moral causes, including domestic trouble, religion, and mental anxiety; 22 to intemperance in drink, and 48 to hereditary influence. Much has been done of late years to remove the bad and erroneous ideas which the general public hold about asylums, and more enlightened views are beginning to prevail; but Dr. Miller says this point should be more strongly urged. He thinks that many of the lower classes are still unwilling to send their friends to an asylum; and there is no doubt that delay in placing lunatics under proper treatment very often results in chronic insanity. We learn with surprise that the Warwick Asylum is not provided with separate infirmaries. Probably it is the only county asylum in England which is not so provided, and we trust the visitors will see their way to at once carry out Dr. Miller's ideas in this respect. Such additions to the institution are not only desirable, but they are essential to careful treatment. Fortunately the asylum has been free from infectious disease during the year. The dysentery prevalent in former years has not reappeared. We do not wish to disturb Dr. Miller's equanimity, but we would remind him that this asylum-dysentery, as it may be called, has a curious way of turning up when it is least expected, and that its dormant period cannot by any means be predicted. Sixty-three per cent. of the nursing staff are in possession of the medico-psychological certificate. Dr. Miller has promulgated a law which will have a stimulating effect on the rest-and-be-thankful section of the attendants; and every member of this section, which we hope is a small one, will see the poetic justice of the law, for no increase of wages and no promotion can be obtained without possession of the certificate. Mrs. Hobbis and Attendants Taylor and Rogers are to be congratulated on obtaining their pensions. The weekly charge for maintenance is 8s. 9d. a head.

### THE KIRKLANDS ASYLUM, GLASGOW.

This is a small asylum, containing only 200 patients on the last day of the year. There were 32 admissions, 16 recoveries, and 19 deaths. The recoveries were 48·4 per cent. of the admissions, and the deaths 7·9 on the average number resident. Of the deaths 7 were caused by cerebral and spinal diseases; 1 from scarlet fever. Consumption was altogether absent from the list of deaths, but intestinal tuberculosis appears once. Dr. Skeen attributes this immunity to the excellence of the diet, to the open air life, and to the fact that when any patient shows signs of tubercle he is treated by isolation just as in any other infectious disease. Scarlet fever broke out, and unfortunately Dr. Skeen was among those who caught the disease, and as there is no assistant medical officer a deputy had to be specially appointed. How the infection reached the asylum is unknown. The weekly rate of maintenance was 8s. 6d. a head.

### THE ROYAL ASYLUM, PERTH.

On January 1st, 1898, this asylum contained 112 patients, and the number had risen to 119 on the last day of the year, not including voluntary boarders. There were 36 admissions, 5 readmissions, 11 recoveries, 15 discharged relieved, and 7 deaths. Calculated on the admissions, the recoveries stand at 27·80 per cent., and the deaths at 5·68 per cent. on the average number resident. Both of these percentages are low, but in so small an asylum one year's statistics are practically valueless. The general average of recoveries since the opening of the asylum stands at 39·14 per cent., and the deaths at 5·33. Of the year's admissions, 14 owed their insanity to various moral causes, and 8 to intemperance in drink. There was one death from pneumonia, but none from consumption. When sufficiently searching inquiries can be made, and correct answers obtained, it is rare to find that only one cause has been at work in the production of insanity. Dr. Urquhart dissects the drink cases, and finds that in only two instances was intemperance the sole cause, and he truly says: "We must discriminate between such as are merely vicious and such as are organically defective in self-control. The fallacy of comparing the expectation of life in individuals who are total abstainers and in those who are not is evident when we know how many weaklings fall victims to alcoholism in the vain endeavour to improve their constitutions and to fit themselves for daily work by constant stimulation." The evils of alcohol are so great that no one need attempt to magnify them; but is it not probable that some of those hereditary cases mentioned by Dr. Urquhart would have remained sane had they been abstainers? Nurse Lawrence won the Morison prize and medal, and six of the taff obtained the Nursing certificate of the Medico-Psychological Association. The aggregate payments of the patients amount to £10,893 per annum, being an average of £83 3s. each, but several patients were maintained at rates varying from £30 to £52 a year.

### THE LANCASHIRE COUNTY ASYLUM AT RAINHILL.

On January 1st, 1898, this asylum contained 1,839 patients, and by the end of the year the number had risen to 2,060. There were 578 first admissions, 47 readmissions, 145 recoveries, 11 discharged relieved, and 239 deaths. Calculated on the admissions of the year, the recoveries stand at 23·20. This is a very low recovery rate, and Dr. Wiglesworth explains it by saying that the asylum is filling up rapidly, and is "made the receptacle for all the wreckage of the district." Calculated on the average number resident the death-rate stands at 12·21 per cent. Of the total number of deaths 84 were caused by cerebral and spinal diseases (and of these 84 we notice that 63 were from general paralysis), 56 were from consumption, 20 from pneumonia, 2 from pleurisy, 2 from empyema, and 2 from colitis. Of the year's admissions 61 owed their insanity to moral causes, 186 to intemperance in drink, and 101 to hereditary influence. As regards the drink cases, we are told that doubtful cases were excluded, and that the figures rather under-estimate than over-estimate the actual facts; and Dr. Wiglesworth adds that "these figures, bad as they are, do not represent the whole extent of the drink evil, for it is undeniable that drinking habits in the parents tend to produce in the children a condition of nervous instability which often culminates in epilepsy or insanity." The weekly cost of maintenance is 8s. 9⅝d.

### LANCASHIRE COUNTY ASYLUM AT LANCASTER MOOR.

This asylum contained 2,068 patients on January 1st, 1898, and the number had risen to 2,122 on December 31st. There were 345 first admissions, 33 readmissions, 132 recoveries, 30 discharged relieved, and 149 deaths. The recoveries were 34·92 per cent. of the admissions, and the deaths were 7·12 per cent. of the average number resident; both of these percentages being below the average. Of the deaths 39 were caused by cerebral and spinal diseases, 22 by pneumonia, 17 by consumption, 2 by enteric fever, and 3 by enteritis. Of the year's admissions 52 owed their insanity to various moral causes, such as domestic trouble and mental anxiety, 27 to intemperance in drink, 38 to hereditary influence, and 78 to previous attacks. As to the latter cause, Dr. Cassidy says it "consists partly in the physical impairment left by the strain of disease on the delicate nerve tissues, and partly in the moral effect produced by the consciousness of a past breakdown, kept alive too often by injudicious friends and the dread of a repeated attack." This is very well put, and it shows once more what a complex thing the causation of insanity really is. The machinery of our county asylums works so smoothly that the public hears but little of what goes on, and hardly comprehends its difficulties. Among the admissions were a man with a fractured rib; another with a broken leg "badly set in plaster of Paris"; another with a cut throat and punctured wounds over the region of the heart; and another acute maniac had recently had part of the lower jaw removed. These cases show what difficult cases asylum nurses have often to take care of. The asylum is inconveniently crowded, and some of the workshops have to be used as dormitories. Classes for the instruction of the nurses were held by Drs. Blair and Erskine, and 21 of the nurses obtained the certificate of the Medico-Psychological Association. The weekly cost of maintenance is 8s. 7⅝d. per head.

